# Wound healing potential of Vakeri fortified *KampillakadiTaila*

**DOI:** 10.1016/j.jaim.2023.100721

**Published:** 2023-05-26

**Authors:** Pranjali P. Dhawal, Milind Gharpure, Minal S. Joshi, Rummana R. Khan, Sidhivinayak S. Barve

**Affiliations:** a Animal Biotechnology and Biochemistry Division, Kelkar Education Trust's Scientific Research Centre, V. G. Vaze College Campus, Mithagar Road, Mulund East, Mumbai, Maharashtra, 400081, India; b Thinq Pharma Cro Ltd A30, Rd Number 10, Wagle Estate, MIDC, Thane West, Thane, Maharashtra 400604, India; c Microbiology Divison, Kelkar Education Trust's Scientific Research Centre, V. G. Vaze College Campus, Mithagar Road, Mulund East, Mumbai, Maharashtra, 400081, India; d Kelkar Education Trust's Scientific Research Centre, V. G. Vaze College Campus, Mithagar Road, Mulund East, Mumbai, Maharashtra, 400081, India

**Keywords:** *Kampillakadi Taila*, Wound healing, Scratch assay, Vakeri, DPPH, Antimicrobial

## Abstract

**Background:**

Traditional medicine in form of decoctions has been known for ages to possess wound healing abilities. One such traditional formulation mentioned in Indian literature Charak Samhita Chikitsa Sthanam is *Kampillakadi Taila* and tremendous information is available on its implication in the treatment of skin cuts and wounds, diseases, or bacterial infections. This research paper focuses on studying the wound healing property of one such herbal proprietary formulation known as a wound healing oil, derived from *Kampillakadi Taila* fortified with root extract of *Wagatea spicata* (VIKHPF).

**Objective:**

The current research is aimed at studying chemical profiling, antioxidant activity, antimicrobial efficacy, *in-vitro* cell proliferating, and *in-vitro* wound healing activity of this VKHPF.

**Materials and methods:**

The chemical characterization of VKHPF was done by gas chromatography- fatty acid methyl esters GC-FAME analysis for lipid analysis and gas chromatography high-resolution mass spectrometry (GC-HRMS)for revealing its chemical constituents. Proliferation and migration are two underlying mechanisms involved in the healing of wounds. Hence, *in-vitro* studies such as cell proliferation assay and *in-vitro* scratch test on NIH/3T3 mice fibroblast cell line were conducted were used to determine *in-vitro* wound healing capacity of VKHPF. The oil was also tested for antioxidant effect (DPPH assay) and anti-microbial potential (Time kill test).

**Results:**

The GC-HRMS and GC-FAME analyses revealed rich medicinally important fatty acids and vitamins were present in VKHPF, such as oleic acid, hexadecanoic acid, squalene, α, γ-tocopherol, γ-sitosterol, and benzoic acid. VKHPF at 0.5 mg/ml in media without serum showed 164.00 ± 0.011% cell viability with 64.00% cell proliferation in contrast to media containing serum (100% cell viability). At the same concentration, the wound closure was 98% for VKHPF. The oil sample possessed antioxidant activity with an IC_50_ value of 3.5 mg/ml and antimicrobial activity against *Staphylococcus aureus* and *Pseudomonas aeruginosa* when tested using Time Kill Activity.

**Conclusion:**

This study is the first to report the use of Vakeri fortified *Kampillakadi Taila* herbal proprietary formulation (VKHPF) in *in-vitro* wound healing and the present data suggest that it can form a part of modern medicine.

## Graphical abstract

**Figure:**
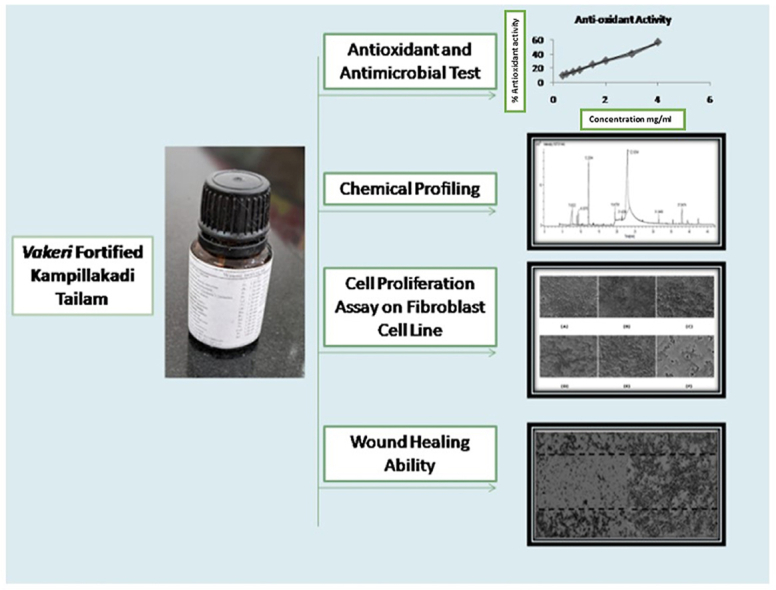

## 1 Introduction

Wound healing occurs in four precise and programmed phases: hemostasis, inflammation, proliferation, and tissue remodeling [1]. The primary goal in treating wounds is rapid wound closure by restoration of structure and functions [2] followed by aesthetically satisfactory scar formation [3]. The role of growth factors [4], chemokines, and cytokines during tissue formation has been the major research focus on the subject of wound healing [5]. A complex signaling network between chemokines, cytokines, and growth factors [6] is important in cell migration, proliferation, and matrix production [7].

Prolongation or failure in one phase may affect the phases of wound healing leading to delay in the healing process or non-closure of the wounds [8]. Alterations in the interplay between phases of wound healing result in chronic wounds. Additionally, excessive deposition of collagen gives rise to hypertrophic scars and keloids [9]. Estimations of chronic wounds are 120 per 0.1 million in the age group 45–65 to 800 in 0.1 million above age 75 years [10]**.** Apart from this, risk factors further responsible for a delayed wound healing process involve age [11], smoking [12], bacterial infection [13], psychological stress and stress-related defects, and other co-morbidities [14]. The management of wounds includes the use of moisture-retentive dressings, topical therapy, and typically disease-specific approaches [15]. For ages, plant-based extracts are considered a promising alternative for wound healing agents by virtue of their diverse secondary metabolites, easy access, and fewer side effects [16]. The idea of exploring plant-based alternatives to modern medicine is to overcome limitations such as high costs, adverse side effects, and long manufacturing times [17].

Herbal remedies, the use of animal or insect products, and the use of organisms to remediate wounds are some traditional forms of wound healing measures adopted by rural people. The types of wounds associated with rural people are ulcers, burns, injuries due to working in fields, conflicts, or traffic accidents. Since such types of injuries fall out of the scope of the World Health Organization (WHO) priority of diseases, traditional medicines have received little attention [18]. As mentioned earlier, the use of traditional plant-based extracts, decoction, or pastes has been practiced in many countries such as India [19,20], Peru [21], and West Africa [22,23]. Plant-based medicines, dating back to 5000 years, are being rediscovered for their human health benefits by modern scientific and evidence methods [24]. Multiple types of research suggest the use of plant extracts in the healing of wounds tested in animal models, viz; *Portulaca oleracea* L in *Mus musculus* JVI-1 [25], a cream containing extract of *Sphaeranthus indicus* on dermal wounds in Guinea pigs [26], asiaticoside isolated from *Centella asiatica* [27], polysaccharides from *Opuntia ficus-indica L. cladodes* on the healing of dermal wounds in the rat [28], oleanolic acid derived *Anredera diffusa* for treating wounds in rats [29] and *Vernonia scorpioides* extract in ameliorating wounds in guinea pig [30]. A series of such phyto-drugs, single or mixed, isolated from plant sources having wound healing properties, are mentioned under the term Vranaopkara in Ayurveda [31].

Surange and Deokule mentioned the use of roots of *Wagatea spicata* (Vakeri) in the treatment of tuberculosis and pneumonia in the Indian Medicinal System and *kampillakadi taila* is mentioned for its application in skin diseases including wound healing [[32], [33], [34]] and ulcers management [35]. A study conducted by Ramesh and Shivram mentions that *Kampillakadi Taila* prepared as per Sharangdhar Samhita, cured 93.33% of cases of wounds in patients within 21 days without any adverse effects. However, the study excluded patients with chronic and traumatic wounds as well as patients suffering from infections like tuberculosis, varicose veins, or co-morbid situations such as diabetes [36]. In yet another study conducted by Nayak and coworkers, *Patolyadi Kwath* and *Kampillakadi Taila* were used to study its wound healing activity on traumatic wounds up to 8 cms in size with exclusions such as stabbed, punctured, surgical and infected wounds. The study revealed in comparison to sterile dressings (5.38%) or *Patolyadi Kwath* (14.89%) used alone, *Kampillakadi Taila* and *Patloyadi Kwath* (69.33%) together showed a significant effect in reduction of the size of the wound within 7 days [37]. The above-mentioned studies emphasize the ability of *Kampillakadi taila* used alone or in combination with vakeri root extracts in wound healing, however, the *in-vitro* data supporting the claim is lacking.

This study is the first to report the *in-vitro* wound healing capacity of Vakeri fortified *Kampillakadi* *Taila* (Table 1), a herbal proprietary formulation (VKHPF). Two major mechanisms involved in wound healing are proliferation and migration of the cells. The VKHPF was tested *in-vitro* for its proliferating capacity using the MTT assay and the cell migration was tested using a scratch test model. The oil sample was also screened for its antioxidant and antimicrobial ability. The gas chromatography (GC) for identification of total fatty acid methyl esters (FAME) and GC high-resolution mass spectrometry (GC-HRMS) profiles revealed components present in the oil sample that can validate its antimicrobial, antioxidant, and wound healing potential. To the best of our knowledge, the Vakeri root extract was a valuable addition to *kampillakadi taila* and this is the first time that the combination has been screened extensively *in-vitro*. Additionally, the oil is being tested on human volunteers, a CTRI registered clinical trial (CTRI/2019/09/021095), for testing its clinical benefits.

**Table 1:** Ingredients in Vakeri Fortified *Kampillakadi Taila*

Sr. No	Ingredients	Botanical names of plant utilized
1.	Vayvidanga (powder of seeds)	*Embelia ribes* Burm.f*.*
2.	Kampillaka trichomes/hair on the surface of fruits	*Mallotus philippensis* (Lam) Muell Arg*.*
3	Kutaj root, bark and stem	*Holarrhena antidysenterica* (L). Wall. ex. A DC.
4.	Trifala seed coats of plant species named	*Emblica officinalis* Gaertn. *or Phyllanthus emblica* L.*, Terminalia bellirica* (Gaertn.) Roxb*., Terminalia chebula* Retz.
5.	Bala oot	*Sida cordifolia* L.
6.	Patol Patra root, fruit, and leaves	*Trichosanthes dioica* Roxb.
7.	Nimsal stem and bark	*Azadirachta indica* A. Juss
8.	Lodhra stem and bark	*Symplocos racemosa* Roxb.
9.	Nagarmotha bulbous part	*Cyperus rotundus* L.
10.	Charolaya seed	*Buchanania lanzan* Spreng.
11.	Khadir sal (stem)	*Acacia catechu* Willd.
12.	Dhayati ful (flower)	*Woodfordia fruticosa* (L.) Kurz
13.	Sarjara resins	*Shorea robusta* Roth.
14.	Agaru stem	*Aquilaria agallocha* (Lour.) Roxb.
15.	Chandan powder of fragrant core of stem	*Santalum album* L.
16.	Sesame Oil	*Sesamum indicum* L.
17.	*Vakeri* root bark	*Wagatea spicata*

## 2 Materials and materials

### 2.1 Materials

Dulbecco's Modified Eagle's Medium (DMEM) was procured from Himedia Laboratories, Mumbai, India. Fetal Bovine Serum (FBS) was purchased from Gibco, Thermo Fischer, USA, 1IU/ml Penicillin and 100 μg/ml Streptomycin were purchased from Himedia Laboratories, Mumbai, India, T25 Flask, and 96 well plates were purchased from Nunc, Nalgene Nunc, International, Rochester, NY, USA. 1X trypsin–EDTA 0.25% was procured from Gibco, Thermo Fischer, USA. Tryptic soy broth was purchased from Himedia Laboratories, Mumbai, India. BD Difco™ Letheen broth was procured from Thermo Fisher Scientific, USA. The bacterial cultures *Staphylococcus aureus* ATCC 6538 and *Pseudomonas aeruginosa* ATCC 9027 were obtained from Kwick-Stik™, Microbiologics, USA. All the other chemicals used were of analytical grade. The sample under study is a polyherbal proprietary Ayurvedic topical formulation used for non-healing wounds manufactured under the trade name D2PRO by ThinQ Pharma Cro Pvt Ltd.

### 2.2 Chemical analysis of VKHPF oil

GC-FAME analysis of VKHPF oil sample was carried out at Geo Chem Laboratories Pvt Ltd. GC-HRMS analysis of the oil sample was carried out at IIT Bombay on Agilent series of GC–MS equipped with headspace injector and Combipal Autosampler and Joel Mass Spectrometer. The ionization energy used was 70 eV, equipped with a time-of-flight analyzer with a mass range of 10–2000 amu and a flame ionization detector (FID). The components identified were compared for their spectra with NIST (National Institute of Standards and Technologies, Mass Spectra Libraries) library.

### 2.3 Cell culture and maintenance

NIH/3T3 cell line was procured from NCCS Pune. Cells were maintained in DMEM and 10% FBS at 37 °C and 5% CO_2_. Media was replenished every alternate day and the cells were trypsinized twice a week. Trypsinization was done with 1 ml 1X Trypsin for 2 min. The cells were centrifuged for 5 min at <1500 rpm. The cells were counted with the aid hemocytometer slide using 0.4% Trypan Blue. For proliferation and wound healing assays, the cells were seeded with known cell densities. The experiments were conducted within five cell passages.

### 2.4 *In-vitro* cell proliferation assay

The herbal proprietary formulation (VKHPF) was screened for cell viability and toxicity on NIH/3T3 fibroblast cell line. Cells were trypsinized and seeded in a 96 well-plate at a density of 0.5 × 10^4^ cells/well in DMEM with 10% FBS. The cells were incubated at 37 °C and 5% CO_2_ overnight for cell attachment. The next day, the media was removed and the cells were treated with different concentrations of 100 μl of VKHPF and sesame oil (0.125, 0.25, 0.5, and 1.0 mg/ml in DMSO), sodium lauryl sulfate (negative control), and dimethyl-sulfoxide (vehicular control) in the serum-deprived condition in DMEM and the plate was incubated at 37 °C and 5% CO_2_ for 24 h. The experiment was conducted in triplicates. The treated 96 well-plate was subjected to quantification of cell viability using MTT assay. The media was removed from each well and 100 μl of 0.5 mg/ml of MTT was added. The plate was incubated at 37 °C and 5% CO_2_ for 4 h. The dye was removed and the formazan crystals formed in live cells were extracted by adding 200 μl of DMSO to each well. The plate was then incubated for 30 min at room temperature in dark. Finally, the plate was read at 530 nm in an ELISA plate reader (MRX Revelation, Thermo Lab Systems).

### 2.5 *In-vitro* wound healing assay using scratch test

Cells were trypsinized and seeded in a 6 well-plate at a density of 2–5 x 10^4^ cells/well in DMEM with 10% FBS. The cells were incubated at 37 °C/5% CO_2_ overnight for cell attachment. The next day, the media was removed and a scratch was made using a 200 μl micropipette tip in each well. The wound was washed using serum-deprived DMEM media twice. The cells were treated with 1.2 ml of 0.5 mg/ml and 1 mg/ml of VKHPF. Serum-deprived media was used as a control. The plate was incubated for 16 h. Photographs were taken at 0 h before treatment and 16 h after treatment. The percent wound healing activity of VKHPF in comparison to media without serum was calculated using ImageJ software. From the images captured, the areas of the scratch (A) were measured using ImageJ software at 0 h and 16 h each. The decrease in the area at different time points indicated percent wound healing activity. The percentage of wound healing activity was calculated by the formula, (A_0_ - A_16_/A_0_)∗100 where A_0_ and A_16_ are the areas of the wound at 0 h and 16 h respectively.

### 2.6 Antioxidant activity

The free radical scavenging ability of VKHPF was analyzed using DPPH (2, 2-diphenyl picrylhydrazyl) assay. DPPH was prepared in methanol at a concentration 10^−6^ M. The VKHPF and sesame oil sample was prepared in methanol to give a final concentration of 0–4 mg/ml. The test solution was prepared by adding 0.5 ml of VKHPF and 0.5 ml of DPPH. Appropriate color blanks were set for the sample. Negative control was prepared by adding 0.5 ml of DPPH and 0.5 ml of methanol. All the tubes were placed in the dark for 30min and the absorbance was read spectrophotometrically at 517 nm. The radical scavenging activity was calculated using the formula (A_NC_-A_T_)∗100/A_NC_, where A_NC_ is the absorbance of negative control, A_T_ is the absorbance of sample or standard test minus absorbance of the respective color blank. Ascorbic acid was used as a standard to determine the ascorbic acid equivalent antioxidant capacity present in 0.5 mg/ml of the oil samples. Additionally, the IC_50_ value of the oil sample was also determined using a standard curve plotted using percent antioxidant activity on the Y-axis versus concentrations of VKHPF on the X-axis.

### 2.7 Antimicrobial assay

The oil sample was tested for antimicrobial ability against *S.aureus ATCC 6538* and *P.aeruginosa ATCC 9027* using a time-kill assay. The sample at 0.5 mg/ml in DMSO was inoculated with 24 h old culture with a density of 10^8^ cfu/ml of both *S.aureus* and *P.aeruginosa* in Tryptic Soy Broth at 37 °C. An aliquot was removed at 4 h and 8 h and was serially diluted in Letheen broth and plating was done on Tryptic Soy Agar. The plates were incubated for 48 h at 37 °C. The colony counts obtained were converted into CFU/ml and the percent kill was calculated for both the culture at respective time points in comparison to the 10% DMSO control.

### 2.8 Statistical analysis

Two-Tailed Unpaired T-test was calculated using GraphPad Prism for the assays conducted for VKPHF formulation. A p-value less than 0.05 was considered to be statistically significant.

## 3 Results

### 3.1 Chemical analysis

**Figure 1:**
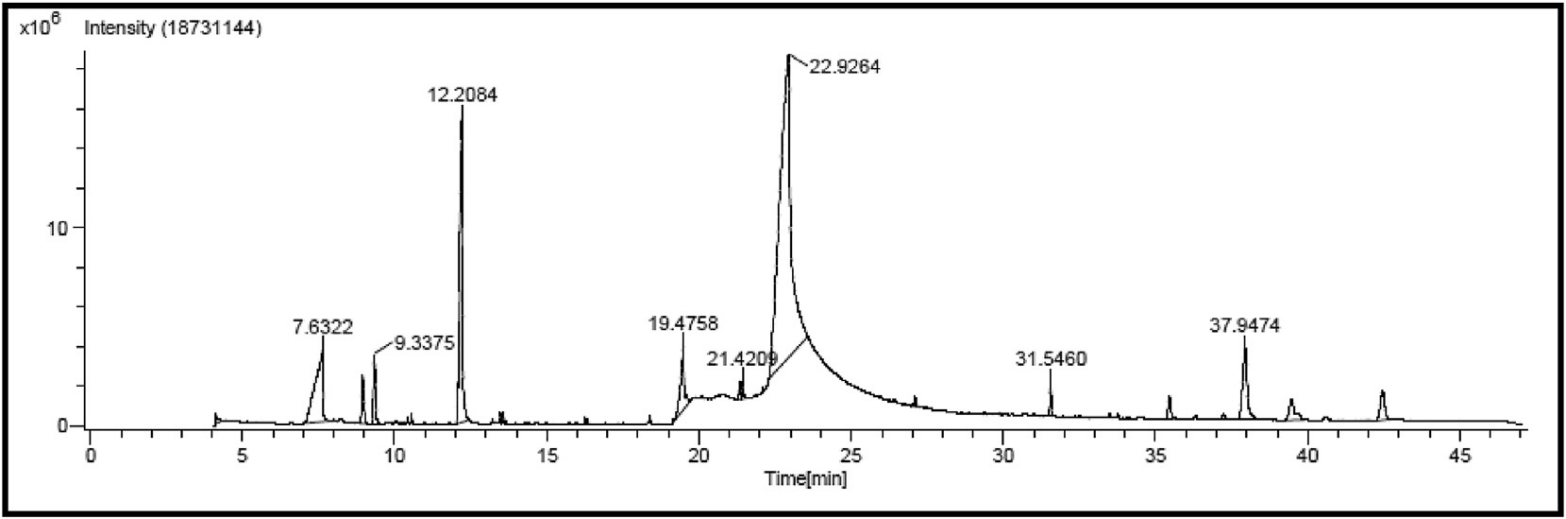
GC-HRMS spectra of VKHPF.

The GC analysis of fatty acid methyl esters present in VKHPF is mentioned in Table 2. Since sesame oil is the major ingredient in the formulation VKHPF, linoleic acid, a major polyunsaturated omega-6 fatty acid was found to be abundantly present in the VKHPF oil sample, followed by oleic acid, palmitic acid, stearic acid, arachidic acid, and eicosenoic acid. The GC-HRMS analysis revealed compounds present in sesame oil. However, certain other peaks were not associated with the common phytocompounds present in sesame oil. The compounds present in VKHPF revealed by GC-HRMS profiling were methyl 2- butanol, benzoic acid, 2,4-decadienal, 3-tert-butyl-4-hydroxyanisole, n-hexadecanoic acid, oleic acid, 9,12-octadecanoic acid, fumaric acid, squalene, γ-tocopherol, dl-α vitamin E, 2,6-bis(3,4 methylenedioxyphenyl)3,7dioxabicyclo [3.3.0] octane, 1,2,5,5,8a-pentamethyl-1,2,3,5,6,7,8,8a-octahydronaphthalen-1-ol, and γ-sitosterol (see Fig. 1).

**Table 2:** GC-FAME profile of VKHPF.

Sr. No	Fatty Acid Profile	Percent in VKHPF
1	C16:0 Palmitic Acid	12.2
2	C18:0 Stearic Acid	2.1
3	C18:1 Oleic Acid	36.5
4	C18:2 Linoleic Acid	47.2
5	C20:0 Arachidic Acid	1.4
6	C20: 1 Eicosenoic Acid	0.2

### 3.2 VKHPF increases cell-proliferation *in-vitro* in NIH/3T3 fibroblast cells

**Figure 2:**
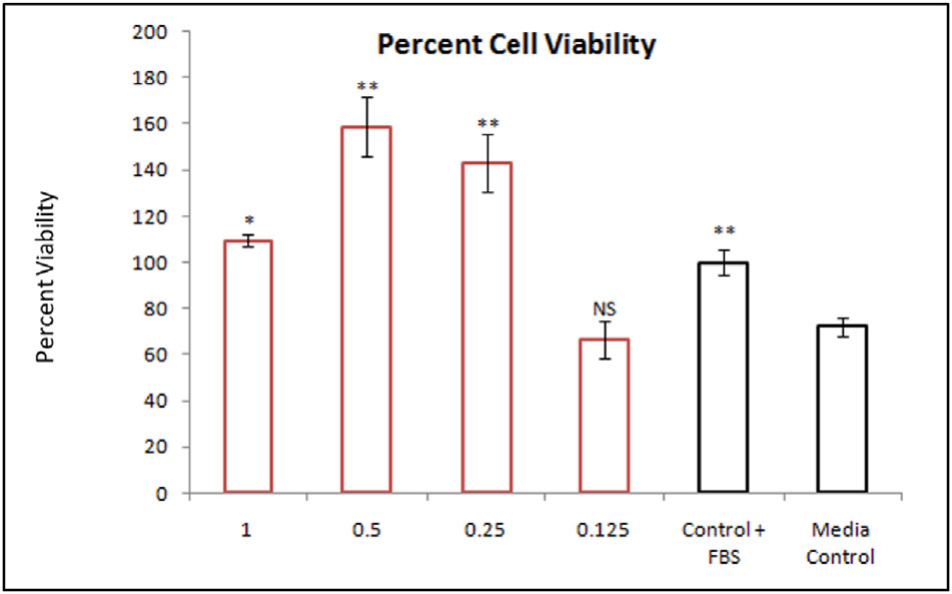
Cell Viability on 3T3 fibroblast cell line.X-axis represents different concentrations of VKHPF that were added (0.125, 0.25, 0.5, and 1.0 mg/ml), media control (without serum) and cell growth control (Media with 10% FBS) (The data was analyzed by Student's T-Test with media control (without serum). ∗ Represents p-value <0.05 and ∗∗ represents p-value <0.01.

**Figure 3:**
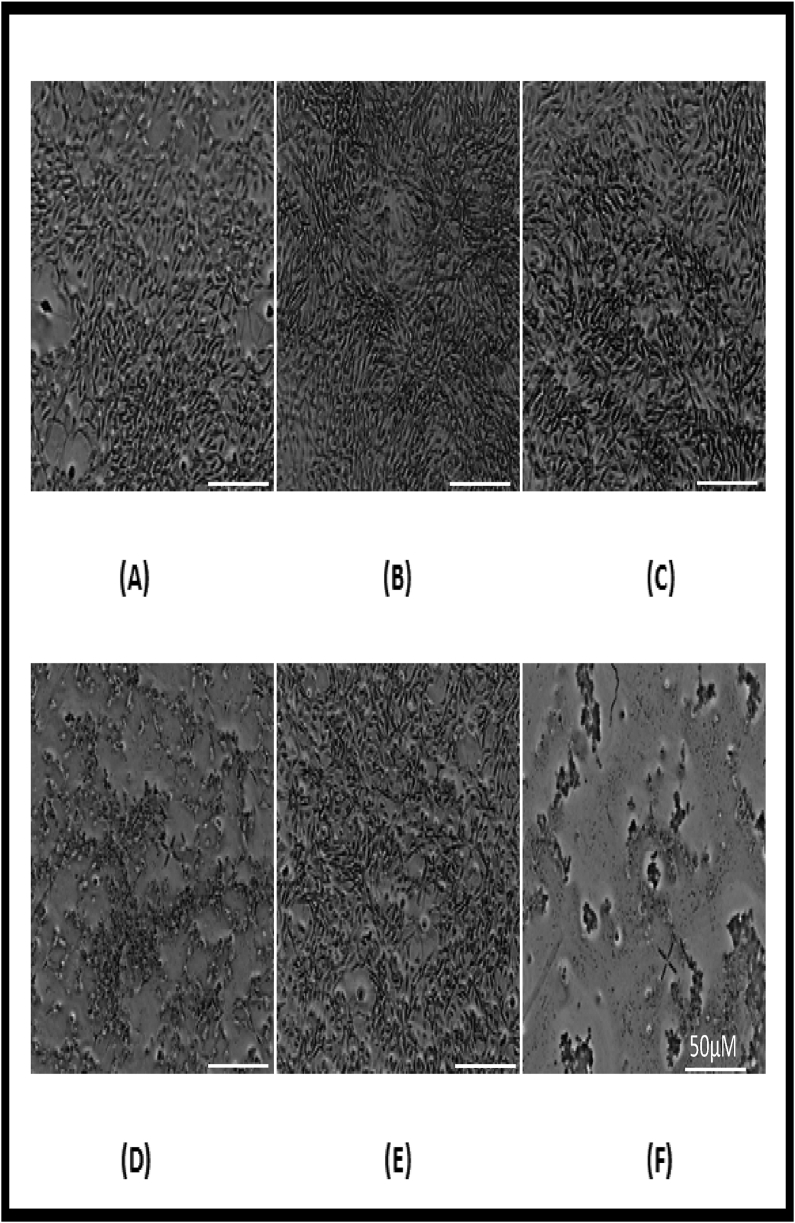
Cell viability assay for VKHPF (A. 1 mg/ml, B. 0.5 mg/ml, C. 0.25 mg/ml, D. 0.125 mg/ml, E. Cell control, F. SLS.

VKHPF was tested for cell proliferation ability on 3T3 mice fibroblast cell lines. The cells were incubated in a serum-deprived medium supplemented with various concentrations of VKHPF (Figure 2, Figure 3). Serum deprivation condition was essential to determine the cell growth-promoting ability of the samples under study. MTT assay was used to determine the cell viability and was measured colorimetrically with a 96-well plate reader. The results showed that up to 1 mg/ml of VKHPF the oil showed no toxic effects in comparison to cells treated with media without serum. In presence of the serum, the viability of the cells was considered 100%, and this was considered to be the growth control for the experiment. In the cells treated with media without the growth factors containing serum, the proliferation was found to be 72.25 ± 0.012%, considered as control media. The cells when treated with 0.125 mg/ml VKHPF also showed similar cell viability of 76.00 ± 0.022% to control media. VKHPF at 0.25 mg/ml showed cell viability higher than media without serum (FBS) and the cell percentage was 88.55 ± 0.025%. However, in comparison to growth control (100%), 0.5 mg/ml of VKHPF showed 164.00 ± 0.011% cell viability where the proliferation was 1.64 times more than growth control. 1 mg/ml of VKHPF showed results similar to the growth control (100%), 106 ± 0.051%. SLS was used as negative control which showed cell viability of less than 10% and DMSO the vehicle control showed negligible effect and cell viability was close to control of media without FBS, 70.88 ± 0.019%. Additionally, sesame oil (0–1.0 mg/ml) alone was also tested for skin proliferation ability, and it was found that the sesame oil did not show any cell proliferation ability. All the tested concentrations of sesame oil showed the same percent cell viability as the media control without serum. Further, the VKHPF concentrations at 0.5 and 1.0 mg/ml were selected for wound healing by scratch test assay. The oil at concentrations of 0.25, 0.5, and 1.0 mg/ml showed a statistically significant increase in the cell growth as compared to cells treated with media without serum (p < 0.01).

### 3.3 VKHPF promoted *in-vitro* cell migration and cell proliferation in NIH/3T3 fibroblast cells

**Figure 4:**
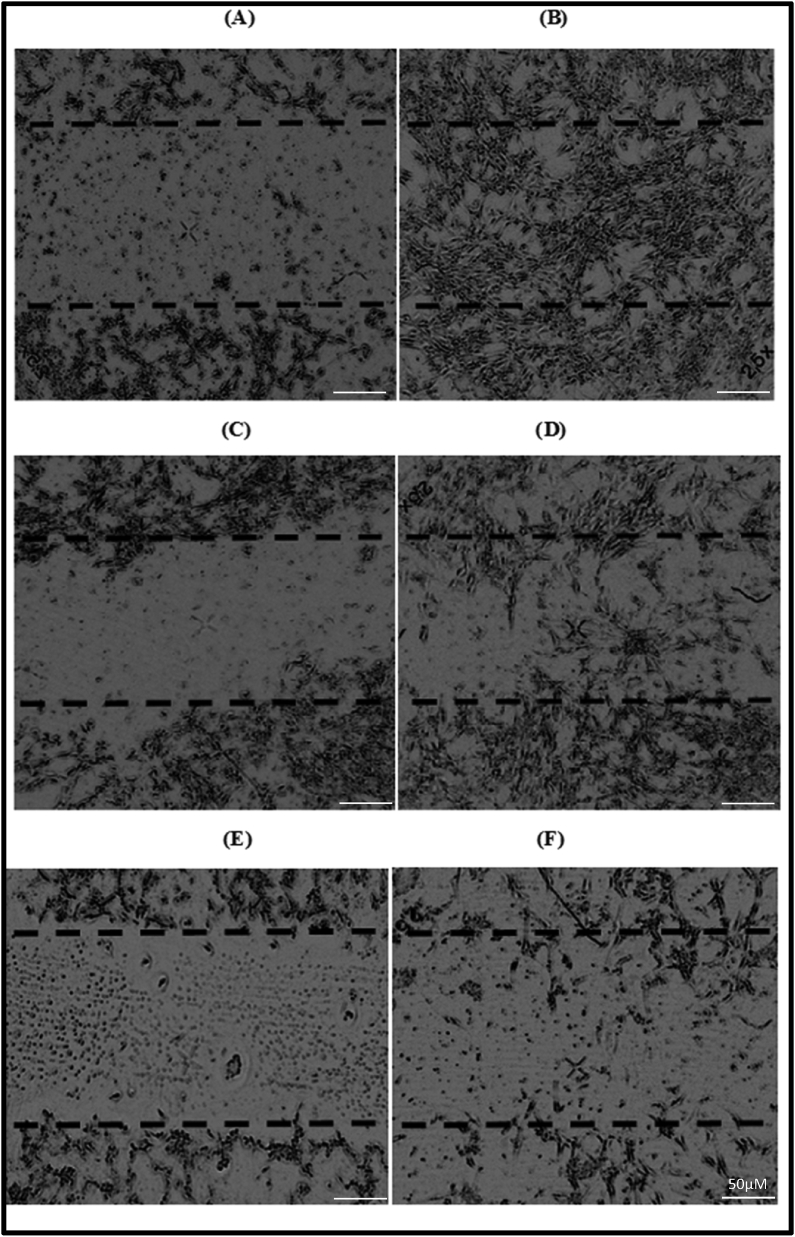
Wound Healing Assay by Scratch Test for VKHPF (A, C, E− 0 h −0.5 mg/ml, 1 mg/ml and media without FBS, B, D, F- 16 h 0.5 mg/ml, 1 mg/ml and media without FBS).

The wound healing ability of VKHPF was assessed using scratch test. The concentrations 0.5 and 1.0 mg/ml of VKHPF were tested *in-vitro* for wound healing ability on the NIH/3T3 mice fibroblast cell line. After 16 h, media without FBS showed minimum proliferation and cell migration and barely healed the scratch. However, for VKHPF the cell proliferation and migration are very evident as seen in Fig. 4. At a concentration of 1.0 mg/ml 47.88% of the scratch was healed by the cell migration capacity of the VKPHF oil sample. However, complete wound healing of 98% was observed in a concentration of 0.5 mg/ml which also co-relates to the highest cell proliferation in the viability test which is 64% more than with 10% FBS (picture not included). The data was found to be statistically significant (p < 0.01) when analyzed using the Students T-Test for both the concentrations tested against cell control.

**Figure 5:**
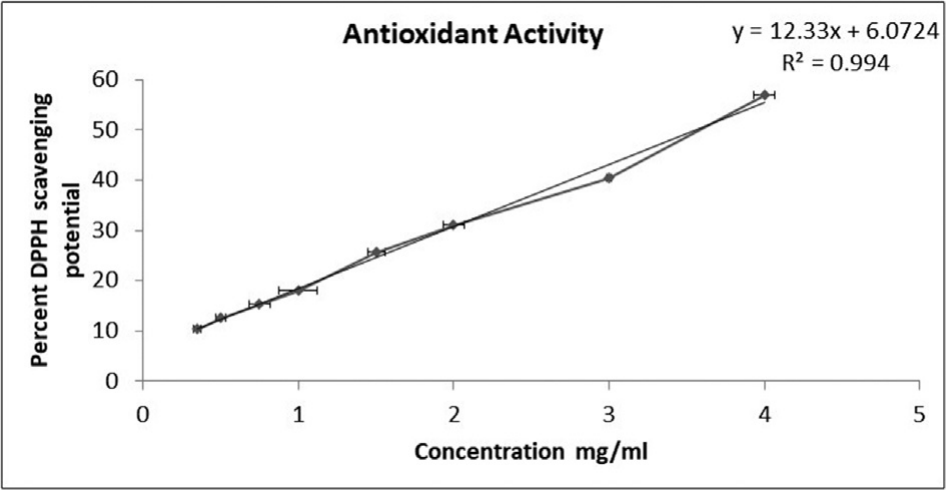
Graph representing Antioxidant Activity of VKHPF oil using DPPH Radical Scavenging Assay.

### 3.4 VKHPF exhibited antioxidant capacity

Since the potent concentration for both proliferation capacity and the scratch test were found to be 0.5 mg/ml, hence, for 0.5 mg/ml, the ascorbic acid equivalent antioxidant capacity was calculated. The VKHPF oil at 0.5 mg/ml has 0.007 mg ascorbic acid equivalent antioxidant capacity. The sample was screened for free radical scavenging/antioxidant ability by DPPH assay at 0.5 mg/ml of the sample. At the tested concentration, 15% antioxidant activity was found in the sample. The IC_50_ value of the sample was found to be 3.5 mg/ml ± 0.21 (see Fig. 5). The oil showed antioxidant potential even at concentrations as low as 0.5 mg/ml. The sesame oil alone showed no antioxidant capacity for the tested concentrations (0–4 mg/ml).

### 3.5 Antimicrobial potential of VKHPF

VKHPF oil sample was tested for its antimicrobial activity against *S. aureus* and *P. aeruginosa* using the Time Kill Method (Table 4). The kill was observed for 4 h and 8 h after allowing the sample to react with respective bacterial species in the tryptic soy broth. The antimicrobial activity was calculated in percentage kill for 0.5 mg/ml of the oil sample tested. Even at such less concentration percent kill was found to be 6% and 15% after 4 and 8 h respectively against *S. aureus.* A slightly higher kill percentage was observed against *P. aeruginosa* which was 11% and 20% after 4 and 8 h respectively.

**Table 4:** Antimicrobial Percent kill activity of VKHPF.

	S.aureus		P.aeruginosa	
	Viable Count		Viable Count	
	4 h	8 h	4 h	8 h
VKHPF Sample (0.5 mg/ml)	48 × 10^4^	33 × 10^4^	57 × 10^4^	48 × 10^4^
Culture Control	52 × 10^4^	71 × 10^4^	125 × 10^4^	193 × 10^4^
% Kill Sample	5.88%	15.38%^a^	10.93%	20%^a^

a Indicates p-value less than 0.05 using Student's T-Test.

## 5 Discussion

The present study tested the Vakeri fortified *Kampillakadi taila* for its cell proliferative, wound healing, antioxidant and antimicrobial capacity. The oil was also tested for the presence of phytochemicals contributed by a series of plant extracts present in the formulation of *Kampillakadi Taila* (Table 1). The phytochemical profiling was done using GC-FAME and GC-HRMS analysis. The data found for GC-FAME analysis is consistent with data present in the literature for oil samples containing sesame oil as the major ingredient [38]. As per the literature, the GC signature of sesame oil reveals the presence of octadecanoic acid, oleic acid, 2,4-decadienal, 3-methyl butanol, γ-tocopherol, and 2,6 Bis(3,4 methylenedioxyphenyl)3,7dioxabicyclo [3.3.0] octane [[38], [39], [40]].

Table 3 represents the GC-HRMS profile for VKHPF. Apart from the lipid compounds present in sesame oil, GC-HRMS revealed the presence of Vitamin E, fumaric acid, sitosterol,1,2,5,5,8a-Pentamethyl-1,2,3,5,6,7,8,8a-octahydronaphthalen-1-ol, benzoic acid, squalene, and vitamin E. The presence of the compounds can be assigned to the varied plant species used for the synthesis of *Kampillakadi taila* [41]. For instance, squalene, sitosterol, and vitamin E are natively present in *W. spicata* [42,43]. Thus, the addition of different extracts in the sesame oil has resulted in increased phytochemicals in the formulation. Many of these compounds can be related to different bioactivities shown by the oil sample in the current study.

**Table 3:** GC HRMS Compounds present in VKHPF and their respective activities.

Sr. No.	Retention Time (in min)	Compound Name	Biological Properties	References
1	4.12	Methyl 2- Butanol	–	–
2	7.63	Benzoic Acid	Antimicrobial agent, used as a preservative	[53]
3	8.94	2,4-Decadienal, (E,E)	Antimicrobial	[54]
4	9.33			
5	12.20	3-tert-Butyl-4-hydroanisole	Antioxidant Agent	[55]
6	19.47	n-Hexadecanoic acid	Antioxidant agent, Anti-androgenic, Hemolytic agent, calming properties	[56]
7	21.32	Oleic Acid	Proliferating properties	[45]
8	21.42			
9	22.92	9,12 Octadecanoic Acid	Anti-inflammatory, antiarthritic, hepatoprotective, antihistaminic	[57]
10	27.08	Fumaric Acid, decyl tetradec-3-enyl ester	Known to have wound healing properties, prevent atherosclerosis, and treat neurodegenerative diseases.	[58]
11	31.54	Squalene	Chemopreventive activity, antioxidant agent	[59]
12	35.44	γ Tocopherol	Anti-inflammatory Activity	[60]
13	37.22	dl-αVitamin E	Antioxidant Agent	[61]
14	37.94	2,6 Bis(3,4 methylenedioxyphenyl)3,7dioxabicyclo [3.3.0] octane	Hyperlipidemic activity, antioxidant, antihypertensive	[62]
15	39.47	1,2,5,5,8a-Pentamethyl-1,2,3,5,6,7,8,8a-octahydronaphthalen-1-ol	Essential Oil Component	[41]
16	42.44	γ-Sitosterol	Induces apoptosis, anticancer agent, and an antiproliferative agent	[63]

For determining the wound healing activity, *in-vitro* cell viability and proliferation assays were used to deduce the limiting cytotoxic concentration of VKHPF. Followed by this, the scratch assay was performed to determine the effect of proliferative concentration of the sample in the induction of cell migration and wound healing. *In-vitro* models for wound healing are used to study re-epithelization, contraction, and angiogenesis [44]. Since sesame oil forms the base of the current VKHPF formulation, it becomes essential to overview the proliferating activity of sesame oil. For ages sesame oil has been used in ayurvedic topical formulations and the slight wound healing activity of sesame oil can be due to the presence of oleic acid. However, sesame oil may not completely heal the wounds, and additional phyto molecules are required to boost its activity. For instance, sesame oil encapsulated rosemary oil and eucalyptus oil nanolipids were tested for *in-vitro* proliferative capacity. Rosemary oil along with sesame oil showed approximately 135% cell viability whereas sesame oil along with eucalyptus oil showed no proliferation. This confirms that cell proliferation was offered by rosemary oil and not sesame oil [45]. Similarly, in the current study, Vakeri fortified *Kampillakadi taila* showed 164% cell viability suggesting the wound healing ability can be assigned not only to sesame oil but also to other phytochemicals added to the oil. It was further confirmed by testing cell proliferative activity of sesame oil which was found to be absent.

*In-vitro* tests are now widely used in ethnopharmacological research for understanding mechanisms related to diseases or the healing process [46]. One such method is wound healing assay using the scratch test, wherein a wound is created on a monolayer of cells, and after treatment, images are captured at regular intervals and compared to quantify the migration, cell–matrix, or cell–cell interactions. It is typically reliable, inexpensive, quick, and mimics migration during the healing of wounds *in-vivo* [47]. Sesame oil along with camphor and honey has been shown to have a better wound healing effect in comparison to control vaseline dressings in mice [48]. In a study conducted by Saporita et al., lipid nanoparticles loaded with essential oils such as rosemary and eucalyptus oil were tested for wound healing ability using the scratch test on fibroblast cell line [45]. Even after 48 h of incubation, complete healing of the scratch was not seen [48]. In contrast to this, complete wound healing of 98% was observed for 0.5 mg/ml of VKHPF, suggesting a synergistic effect of sesame oil along with other phytochemicals present in the formulation of VKHPF.

In the current study, the sample was also tested for antioxidant and antimicrobial capacity. The sample showed an IC_50_ value of 3.2 mg/ml for DPPH radical scavenging ability. The sesame oil alone showed no DPPH scavenging activity till 4 mg/ml suggesting that the antioxidant activity is due to the varied phytochemicals contributed by plant materials apart from sesame oil in the VKHPF formulation. The presence of phytocompounds such as tannins, saponins, terpenoids, cardiac glycosides, phenolics, and flavonoids may result in antioxidant activity of VKHPF and may be attributed to the *Kampill*aka trichomes/hair of the fruit, *W. spicata* roots, and *Sida cordifolia* [49,50].

The antimicrobial activity found for VKHPF was very marginal. However, it serves as an added benefit for the oil with high cell proliferating activity on fibroblasts and radical scavenging activity. Reports suggest that *W. spicata,* when used alone, has revealed antimicrobial activity against methicillin-resistant *S. aureus* [51]. The antimicrobial activity of the oil sample may be attributed to the presence of Vakeri extract along with other phytocompounds extracted from the different plant materials used in formulating VKHPF. Literature also reveals that *Azadirachta indica* is effective against planktonic cells and biofilms caused by *P. aeruginosa* [52] which is a component of the oil sample.

## 5 Conclusion

To summarize, the *Vakeri* fortified *Kampillakadi Taila* showed significant proliferative and wound healing capacity when studied *in-vitro.* Apart from this the other known properties of both *Vakeri* extract and *Kampillakadi Taila* such as antioxidant, anti-inflammatory, and antibacterial, among others suggests that the formulation can prove to be an effective remedy for wound healing. The study thus concludes the ethno–pharmaceutical activity of *Vakeri* fortified *Kampillakadi Taila* with immense potential in the pharma industry.

## Source of funding

None.

## Contributions

All authors equally contributed to the preparation of Manuscript. PPD and MSJ performed the assays. PPD, MG and SSB supervised the study. PPD, MG and RRK wrote the manuscript.

## Declaration of competing interest

The authors declare no conflicting interest.
